# Assessing ChatGPT-v4 for Guideline-Concordant Inflammatory Bowel Disease: Accuracy, Completeness, and Temporal Drift

**DOI:** 10.3390/jcm14134599

**Published:** 2025-06-29

**Authors:** Oguz Ozturk, Mucahit Ergul, Yavuz Cagir, Ali Atay, Kadir Can Acun, Orhan Coskun, Ilyas Tenlik, Muhammed Bahaddin Durak, Ilhami Yuksel

**Affiliations:** 1 Department of Gastroenterology, Ankara Bilkent City Hospital, Ankara 06170, Turkey; dr.mucahitergul@gmail.com (M.E.); draliatay@hotmail.com (A.A.); kacun01@gmail.com (K.C.A.); drcoskunorhan@gmail.com (O.C.); ilyastenlik@yahoo.com (I.T.); yukselilhami@hotmail.com (I.Y.); 2 Department of Gastroenterology, Ankara Yildirim Beyazit University Yenimahalle Training and Research Hospital, Ankara 06560, Turkey; yvzcgr@hotmail.com; 3 Department of Gastroenterology, Faculty of Medicine, Hacettepe University, Ankara 06100, Turkey; docmbd@gmail.com; 4 Department of Gastroenterology, Faculty of Medicine, Ankara Yildirim Beyazit University, Ankara 06110, Turkey

**Keywords:** ChatGPT, inflammatory bowel diseases, artificial intelligence, clinical decision support

## Abstract

**Background/Objectives:** Chat Generative Pretrained Transformer (ChatGPT) is a useful resource for individuals working in the healthcare field. This paper will include descriptions of several ways in which ChatGPT-4 can achieve greater accuracy in its diagnosis and treatment plans for ulcerative colitis (UC) and Crohn’s disease (CD) by following the guidelines set out by the European Crohn’s and Colitis Organization (ECCO). **Methods:** The survey, which comprised 102 questions, was developed to assess the precision and consistency of respondents’ responses regarding the UC and CD. The questionnaire incorporated true/false and multiple-choice questions, with the objective of simulating real-life scenarios and adhering to the ECCO guidelines. We employed Likert scales to assess the responses. The inquiries were put to ChatGPT-4 on the initial day, the 15th day, and the 180th day. **Results:** The 51 true or false items demonstrated stability over a six-month period, with an initial accuracy of 92.8% at baseline, 92.8% on the 15th day, and peaked to 98.0% on the 180th day. This finding suggests a negligible effect size. The accuracy of the multiple-choice questions was initially 90.2% on Day 1, reached its highest point at 92.2% on Day 15, and then decreased to 84.3% on Day 180. However, the reliability of the data was found to be suboptimal, and the impact was deemed negligible. A modest, transient increase in performance was observed at 15 days, which subsequently diminished by 180 days, resulting in negligible effect sizes. **Conclusions:** ChatGPT-4 demonstrates potential as a clinical decision support system for UC and CD, but its assessment is marked by temporal variability and the inconsistent execution of various tasks. Essential initiatives that should be carried out before involving artificial intelligence (AI) technology in IBD trials are routine revalidation, multi-rater comparisons, prompt standardization, and the cultivation of a comprehensive understanding of the model’s limitations.

## 1. Introduction

Inflammatory bowel disease (IBD) is the term used to describe a set of complicated and chronic disorders that present as relapsing and remitting, besides influencing multi-systemic disorders, particularly those presenting symptoms involving the gastrointestinal system. Ulcerative colitis (UC) and Crohn’s disease (CD) are the well-known types of IBD. These disorders are rapidly increasing at concerning rates and are causing a major worldwide public health issue [1]. Despite significant advances in the diagnosis, imaging, and treatment of IBD, both patients and healthcare professionals continue to face challenges, particularly regarding etiology, accurate diagnosis, and treatment costs [2]. Concurrent with these clinical challenges are advancements in artificial intelligence (AI), such as Chat Generative Pre-Trained Transformer (ChatGPT). Large Language Models (LLMs) have been engineered to learn from vast quantities of text and images, thus enabling them to discern patterns and generate new, pertinent content in a manner analogous to human cognition. These developments open up a new era in terms of enhancing longitudinal patient follow-up, broadening access to reliable information, and streamlining complex decision-making processes. ChatGPT employs training based on extensive textual corpora to interpret natural language and generate context-based responses using an algorithm that imitates human cognition [3]. To date, there has been a paucity of studies that evaluate guidelines for IBD-specific questions. However, the majority of the research in this field has focused on the general reliability of ChatGPT and the other LLMs when compared to conventional information sources or healthcare professionals [4,5]. This study depends on prior assessments of ChatGPT-4 in additional gastroenterological contexts. These earlier assessments have demonstrated encouraging results as outlined in publications [6,7].

The present study employed a methodical approach to underscore ChatGPT-4’s capacity to mirror the ECCO guidelines for the clinical decision support for IBD. Specifically, the objective is to quantify factual concordance with guideline statements and ascertain the completeness and readability of ChatGPT-generated responses to common clinical scenarios. The questions contain induction and maintenance therapy, surveillance protocols, and extra-intestinal manifestations, and investigate the potential for these outputs to meaningfully assist clinicians in decision-making in daily practice. It is the objective of this study to methodically assess the merits and limitations of ChatGPT in the field of IBD. Additionally, the study underscored the assessment of the potential functionality of these models as auxiliary tools, rather than as substitutes, by subjecting ChatGPT-4 to a rigorous benchmarking process against globally acknowledged norms.

The objective of our study was to assess the efficacy of ChatGPT-4 in the diagnosis and management of UC and CD. The overarching objective of this investigation was to ascertain the potential utility of ChatGPT-4 as a clinical decision support tool and as a valuable educational resource for clinicians.

## 2. Methods

### 2.1. Study Design and Settings

The present study was conducted between April 2024 and November 2024. A total of 102 questions were developed for the study, designed to simulate real-world clinical scenarios and decisions. The questions were categorized into two types: true or false questions (51 questions) and multiple-choice questions (51 questions). The objective of this study is to assess the ability of ChatGPT to confirm or deny statements in accordance with the ECCO guidelines. The study did not involve human or animal subjects, given that it was exclusively questions formulated by the research team that were subject to evaluation by Chat-GPT; therefore, ethical approval and informed consent were not deemed necessary.

The responses of ChatGPT-4 to the questions were evaluated using a six-point Likert scale (1 = completely false, 2 = mostly false, 3 = false and true are equal, 4 = more true than false, 5 = almost completely true, 6 = true) to assess the accuracy of each response. The degree of completeness of the AI responses was assessed in terms of whether they provided all the necessary information regarding the topic in question and in terms of the level of detail and depth of the responses provided. The level of detail in the responses was evaluated on a 3-point scale (1. Inadequate—the responses only address partial aspects of the question, omitting key elements; 2. Adequate—the response covers all aspects of the question and provides the minimum necessary information, thus meeting the criteria for completeness; and 3. Comprehensive—the response fully addresses all aspects of the question and provides additional information or synthesis that goes beyond what was expected).

The true or false questions were designed to assess ChatGPT’s ability to correctly confirm or deny statements based on ECCO guidelines on IBDs. The multiple-choice questions aimed to assess ChatGPT’s ability to select the correct option among various possibilities, once again based on the aforementioned ECCO guidelines. The preparation of two different types of questions allows for a more objective evaluation of ChatGPT, as the correct and incorrect answers can be easily identified. A reevaluation of the binary and multiple-choice questions pertaining to IBD was conducted at the 15-day and 180-day intervals following the initial and subsequent responses of ChatGPT-4. In order to ensure the replicability of our study and to minimize the impact of conversational bias, we posed each inquiry in a distinct chat session, utilizing the standard settings of the ChatGPT interface.

The rationale for selecting this interval was to enable an assessment of the temporal evolution of ChatGPT-4 responses and to ascertain its capacity for learning. The 15-day interval was selected to evaluate the reliability of short-term responses. The 180-day period was selected to assess long-term model drift, considering the probability of it being less influenced by substantial model version updates. In accordance with the aforementioned theories, 51 multiple-choice questions and 51 true-false questions were posed to ChatGPT. After answering the binary questions as true or false, ChatGPT-4 attempted to provide explanatory information in support of its answer, similar to that required in a multiple-choice question. This enabled us to score in line with the Likert scale.

A further aspect of the evaluation pertained to the AI’s capacity to provide consistent responses to comparable queries. Additionally, the degree of comprehensiveness of the responses were also considered. The AI’s assessment was that the ECCO guidelines were sufficiently comprehensive and detailed. One of the most significant aspects of the study was the reevaluation of responses initially deemed inaccurate by ChatGPT-4 (i.e., those scoring less than three points on the accuracy scale). In order to ascertain the impact of elapsed time on the AI’s accuracy, we decided to implement a continuous update process, which would allow for meaningful data collection. Consequently, a second round of questioning was conducted with ChatGPT-4 15 days later to improve the AI’s accuracy at six months.

### 2.2. Statistical Analysis

The execution of all operations was conducted using IBM SPSS Statistics for Windows, Version 25.0 (developed by IBM Corp., located in Armonk, NY, USA). The ordinal results from the 3- and 6-point Likert scales generated from the original data are presented as median and interquartile range (IQR). Categorical variables are expressed as frequencies and percentages. The Kolmogorov–Smirnov and Shapiro–Wilk tests, substantiated by quantile–quantile plots and histograms, facilitated the assessment of data normality. Given the majority of the variables’ deviation from normality, non-parametric techniques were implemented in succession.

The Friedman test was employed to examine temporal fluctuations across the three measurements: The first phase extends from Day 0 to Day 15. The subsequent phase extends from Day 15 to Day 180. In instances where the omnibus test demonstrated significance, a Wilcoxon signed-rank test was employed in conjunction with a Holm–Bonferroni correction. The effect size for significant Wilcoxon test results was calculated using r = Z/√N. The assessment of overall differences was conducted using Cochran’s Q statistic, followed by McNemar tests (Holm-corrected) for specific contrasts pertaining to binary and multiple-choice accuracy (correct/incorrect). The reliability of the answers over time was measured using the weighted Cohen’s kappa coefficient. No imputation was performed, and all analyses utilized complete case data. A two-tailed *p*-value < 0.05 following Holm’s correction was established as the statistical significance metric. Raw *p*-values are displayed alongside their adjusted counterparts to facilitate comprehension.

## 3. Results

### 3.1. Binary Questions

**Figure 1:**
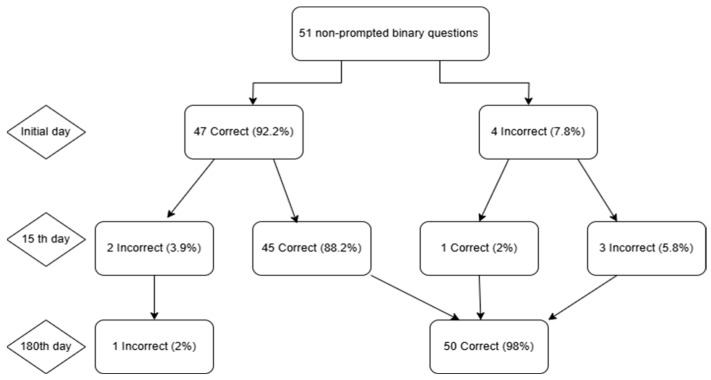
Flowchart illustrating ChatGPT-4′s answers to 51 binary questions across three time points. Day 0 began with 47 correct (92.2%) and 4 incorrect (7.8%) responses. By Day 15, 45 items remained correct, 2 correct answers became incorrect, 1 incorrect answer became correct, and 3 errors persisted. At Day 180, 50 answers were correct (98.0%) and 1 remained incorrect (2.0%), depicting an overall net gain despite interim fluctuations.

The accuracy of the responses generated by ChatGPT-4 to binary queries on IBD was evaluated across three periods: at baseline and at the 15-day and 180-day intervals. The accuracy remained unchanged at 92.2% (47/51) for the 51 questions in binary format at day 0 and at day 15. At day 180, the accuracy increased to 98.0% (50/51). The incorrect answer rates were 7.8%, 7.8%, and 2.0%, respectively. In the initial 15-day period, 3.9% of the questions transitioned from correct to incorrect, 2.0% shifted from incorrect to correct, and 5.9% maintained their original incorrect status. By day 180, there were no more questions that transitioned from correct to incorrect; 7.8% of the previously incorrect questions transitioned from correct to correct, while only 2.0% remained incorrect (Figure 1).

**Figure 2:**
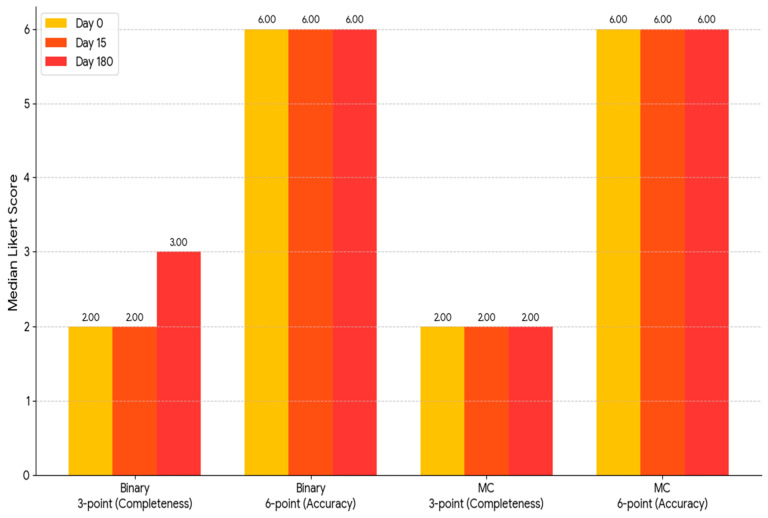
A grouped bar chart displaying the median Likert ratings for “completeness,” with a scale of 3 points, and “accuracy,” with a scale of 6 points, for ChatGPT-4 on Day 0, Day 15, and Day 180 is displayed in Figure 3. The three consecutive evaluations are indicated by yellow, orange, and pink bars, respectively. As illustrated, the two groups of bars on the right display the same scales for the 51 multiple-choice questions. Conversely, the two groups on the left represent the 51 binary (true/false) questions, first in terms of completeness and subsequently in terms of accuracy. As illustrated by the graph, the median scores demonstrated significant stability over time. The sole discernible fluctuation was an increase in the integrity score of the correct/incorrect questions on Day 180. MC: multiple choice.

To assess the precision and thoroughness of the measurements, a comparison of matched samples was extended to three measurement occasions: As demonstrated in Figure 2, the median accuracy score on the 6-point scale assigned to binary questions was invariably 6.0 (with an interquartile range [IQR] of 1.0) across all evaluations. Initially and on Day 15, the median completeness score on the 3-point scale was 2.0 (interquartile range (IQR): 1.0). By the 180th day, the mean value had reached 3.0 (interquartile range [IQR] 1.0), which constituted a statistically significant change (*p* = 0.028). The mid-term fluctuation is attributable to random or transitory variation rather than a lasting deterioration in model performance (Table 1 and Table 2).

### 3.2. Multiple-Choice Questions

**Figure 3:**
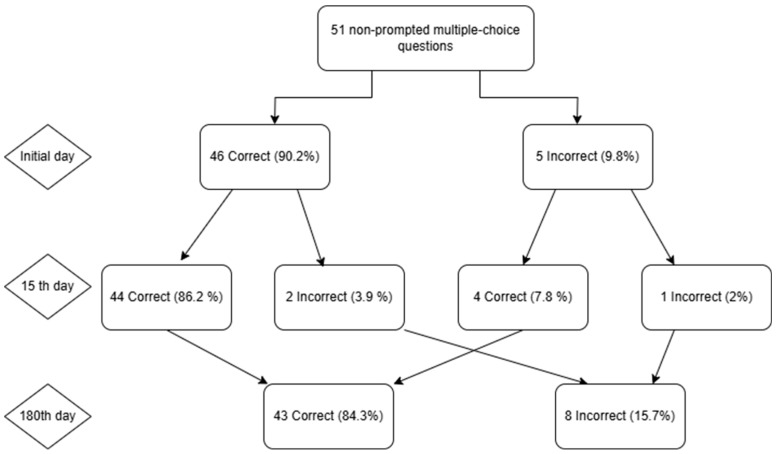
The following flowchart illustrates ChatGPT’s performance on 51 multiple-choice questions over a specified time period. The initial test exhibited five errors, accounting for 9.8% of the total mistakes, while demonstrating an accuracy rate of 46 out of 51, corresponding to 90.2% precision. On the 15th day of observation, the highest proportion of correct answers was observed to be 47 (92.2%). By the 180th day, the number of accurate responses decreased to 43 (84.3%), while the number of incorrect answers increased to 8 (15.7%). This indicates that the transient improvement observed on Day 15 had resolved.

In a series of 51 multiple-choice questions, the accuracy rate of ChatGPT-4 exhibited a consistent increase from 90.2% (46/51) on day 0 to 92.2% (47/51) on day 15, followed by a decline to 84.3% (43/51) on day 180. The percentage of erroneous responses was 9.8%, 7.8%, and 15.7%, respectively. From the initial baseline to the 15th day, a total of 3.9% of the questions exhibited a shift from correct to incorrect, 7.8% demonstrated a transition from incorrect to correct, while 2.0% retained their original accuracy classification. From Day 15 to Day 180, there was a 13.7% increase in the transition from true to false, a 5.9% increase in the transition from false to true, and a 2.0% decrease in the number of responses that remained false (Figure 3).

The analysis of the accuracy of the multiple-choice responses revealed a marked difference across the duration of the study, thereby underscoring concerns regarding the long-term dependability of the model. The median accuracy score on the 6-point scale demonstrated stability at 6.0 for all designated time points, and the median completeness score on the 3-point scale exhibited consistency at 2.0 for all specified time points. While the medians remained constant, the underlying distributions underwent statistically significant changes on Day 15 (*p* = 0.001). This finding indicates that the performance initially reached a peak but subsequently declined after six months. These values were no different from the baseline. Therefore, while ChatGPT-4 exhibited elevated accuracy in multiple-choice outputs at Day 15, the ensuing decline, coupled with a persistent low agreement coefficient (κ = 0.18), suggests that this initial enhancement might not signify an enduring improvement in performance (Table 3 and Table 4).

## 4. Discussion

ChatGPT exhibits a high degree of baseline accuracy in providing correct answers to binary questions (92.2%) and correct answers to multiple-choice questions (90.2%). However, our longitudinal evaluation of ChatGPT-4 in comparison to the binary and multiple-choice items reveals that its performance is subject to variation. On the 180th day of the period, the binary responses were consistent with a significant degree of reliability and improvement. Yet, concerning the multiple-choice responses, a brief surge in accuracy was observed on the 15th day, reaching a percentage of 92.2%, which subsequently declined to a level below the established baseline on Day 180, with an accuracy of 84.3%. The extent of the participants’ agreement with the presented statements was not uniform. The weighted Cohen’s kappa demonstrated a moderate degree of agreement for binary items (0.652). In contrast, for the multiple-choice items, the agreement was found to be insufficient (0.181). This observation aligns with the findings of the ordinal ratings, which documented smaller effect sizes.

The findings of our investigation align with the results of the study by Johnson et al., who recommended a prudent clinical adoption of ChatGPT, given its generally accurate responses to gastroenterological inquiries, though with the caveat that occasional errors occur. By demonstrating the enhancement of absolute performance (the initial 90% accuracy) and the presence of time-dependent drift, it is considered a complement to Sciberras’ ECCO-focused snapshot of ChatGPT v3.5′s accuracy (82.4%) and inclusiveness (78.9%). It is noteworthy that most of the initially accurate responses remained so upon subsequent retesting. Only a small percentage of the responses shifted from accuracy to inaccuracy, thus indicating latent instability that warrants physician observation.

A notable finding was the discrepancy in the median accuracy and completeness scores. A median score of 6.0 on the 6-point accuracy scale indicates that ChatGPT-4 effectively provided factually correct responses in accordance with the ECCO guidelines, with most answers receiving a “true” rating (a score of 6). In contrast, the median completeness score was generally 2.0 on a 3-point scale. According to the scale’s definition, a score of 2 indicates “Adequate”, meaning the response provides comprehensive coverage of all aspects of the enquiry and includes essential information. To attain a score of 3, which denotes “Comprehensive”, it was necessary to provide additional information beyond that requested. Our findings suggest that, while ChatGPT-4 demonstrates a high degree of accuracy, its responses are often adequate but not exhaustive.

Previous research, like that of Lahat et al., employed scaling and evaluated the consistency and accuracy of the treatment of CD and UC [8]. Cankurtaran et al. also referenced this study, noting that the same questions had been posed but that different responses were received and that AI generated distinct answers for each individual [4]. Our study employed a similar methodology to the aforementioned study, namely the administration of a series of identical questions at 15-day and 180-day intervals. This investigation differs from the previous study; the present study’s objective was assessing the temporal consistency and drift in responses rather than the responses themselves.

For the purpose of patient-oriented education regarding IBD, Zhang et al. conducted a comparative analysis of ChatGPT-4, Claude-3-Opus, and Gemini-1.5-Pro. Their findings revealed that all three systems demonstrated a high degree of accuracy. Specifically, ChatGPT-4 was identified as the most accurate in terms of content relevance, while Claude-3-Opus exhibited superior readability. Their trial underscores that the quality of the model decreased for complex queries, a finding that aligns with our observation that multiple-choice questions demonstrated lower stability compared to binary choices [9]. Yan et al. reported that a one-time ChatGPT-4 utilization session can achieve parsimony with clinicians in patient education quality [10]. Ran et al. specifically highlight this evidentiary gap, and Zeng et al. demand time-series data on ChatGPT-4 drift [11,12]. Ghersin et al. reported 87.8% accuracy for 30 clinical scenarios; however, the study was restricted to a single, cross-sectional session [13]. In contrast, Sciberras et al. demonstrated that ChatGPT-3.5 responded to 38 IBD educational inquiries with high apparent accuracy (84.2%), though only one-third of responses were fully complete [5]. Conversely, the probes employed by Kerbage et al. and Naqvi et al. encompassed only a subset of patient searches, with a range of twenty and six, respectively. Despite demonstrating a 75–83% reliability range, these probes also exposed significant content gaps and errors in healthcare viewpoints [14,15].

Hitherto the studies applying ChatGPT to IBD mostly focused on instantaneous performance. Our study addressed this critical gap by longitudinally examining ChatGPT-4 over a period of 180 days. In addition to the instantaneous complexity issues identified by Zhang et al., our study also unveiled model drift over time. This imperative stands in contrast to patient education-oriented methodologies proposed by Yan et al., which prioritize patient education.

In contrast to Gravina et al., who evaluated 10 prevalently patient-centered inquiries over the course of three days using ChatGPT 3.5 and noted superficially accurate yet generally correct responses, Levartovsky et al. evaluated 20 UC cases and observed an 80% concordance with physician decisions in a limited, solitary-time-point sample [16,17]. The aforementioned studies made a scarce mention of the crucial element of assessing ChatGPT’s temporal consistency and its tendency to exhibit a drift in responses. This crucial element was pivotal to the establishment of our study, and it also diverged from short-term or single-assessment research. This comprehensive and long-term approach provided more profound and nuanced insights into topics such as ChatGPT’s progress over time and its potential benefits to clinicians.

Lusetti et al. synthesized eight trial reports and concluded that, despite the encouraging cross-sectional accuracy of the models in terms of supporting decision-making, significant challenges remain, particularly in terms of model consistency, adherence to established guidelines, and the phenomenon of version drift [18]. Given the findings outlined in our study and the challenges identified by Lusetti et al., there emerged a compelling necessity for the routine revalidation of ChatGPT to ensure its continued reliability for clinical practice.

The present study utilized non-parametric statistics with Holm adjustment and effect-size metrics. It employed 102 ECCO-based questions administered at three time points up to Day 180. The study depended on expert raters with significant experience in IBD, thereby enhancing internal validity. The design presented here offers a novel approach to the implementation of ChatGPT in the context of IBD treatment. It provided the first precisely measured, guideline-aligned longitudinal benchmark, which has not previously been observed in this field. A rigorous evaluation of the model’s accuracy was conducted by leveraging the statistical methodology of a weighted Cohen’s kappa and employing expert validation through adjudication. While its answer set evolves over periods ranging from weeks to months, ChatGPT-4 offered prompt and largely precise guidance for the treatment of IBD. The present study underscores ChatGPT’s utility for clinical decision support for clinicians. It is imperative to emphasize the necessity of the periodic revalidation of ChatGPT-4 to ensure its reliability for clinical applications. Within the subgroups, no statistically significant disparities were identified in the accuracy of the responses to the diagnostic, therapeutic, and imaging inquiries at the initial assessment and at the follow-up evaluation. In summary, the responses to these three domains were found to be equally accurate. However, it is not possible to provide a definitive answer at this time. Further analysis may be necessary to achieve a more nuanced comprehension of the issue.

**Figure 4:**
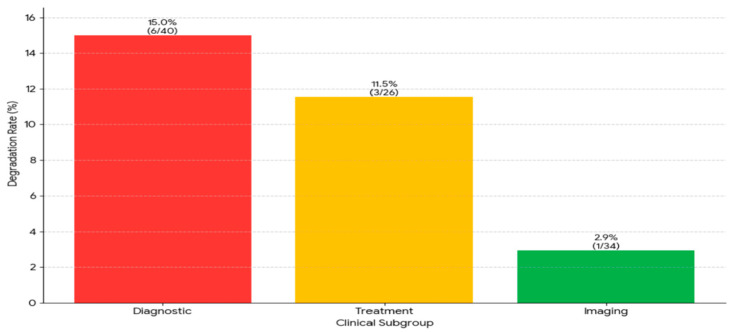
The analysis of response instability in subgroups revealed significant differences between groups. Diagnosis questions had the highest deterioration rate at 15.0% (6 out of 40 questions), followed by treatment questions at 11.5% (3 out of 26 questions). Imaging questions had the highest stability, with a rate of 2.9% (1 out of 34 questions).

We conducted a comprehensive study, examining all instances where an initially correct response subsequently proved to be inaccurate. “Answer degradation” was observed in 10 out of 102 cases during the 180-day period, indicating a frequency of 9.8% within this time span. A marked difference in the degree of stability was evident between the binary and multiple-choice questions. The proportion of binary questions exhibiting instability was only 3.9%, while a substantially higher 15.7% of multiple-choice questions demonstrated such instability, indicating a significant increase in the prevalence of this issue. When the data were disaggregated by clinical subgroup, the types of questions exhibiting the greatest prevalence of this type of degradation were those of a diagnostic nature (6 out of 40, constituting 15.0%) and those concerning treatment (3 out of 26, representing 11.5%). The study’s findings indicate that only a small percentage of the imaging questions exhibited signs of degradation. This observation indicates that the questions exhibited an unparalleled degree of stability. This finding suggests that the temporal instability of the model may not be random. This instability is particularly notable in the context of diagnostic and therapeutic inquiries, particularly when these inquiries are presented as multiple-choice questions (Figure 4).

Although this study had strengths, it also had limitations, first including a single-center design and a limited data pool. Second, although performance metrics were recorded over a period of 180 days, it proved impossible to forestall minor, unannounced modifications to the ChatGPT-4 model’s underlying architecture. These alterations, if any, may have occurred over the course of time and could potentially influence the responses generated by the model. Third, non-parametric statistical tests, including the Friedman test and Kendall’s W, were employed to analyze ordinal data from Likert scaling models. The demonstration that a *p*-value analysis is subject to inaccuracy if there are excessively many ties can be evidenced by the fact that scales with a limited number of points, for example, the 3-point completeness scale, are employed. The results of this study indicate that utilizing more sophisticated scales in subsequent research may be conducive toward a more precise evaluation of the statistical problem. Future studies should include the following: the use of multiple raters, a more extensive question bank, and head-to-head comparisons of various GPT versions over extended follow-up periods.

## 5. Conclusions

The present study demonstrated that ChatGPT-4 had a high level of binary accuracy, with its multiple-choice performance reaching its peak at 15 days and subsequently declining below the baseline by 180 days. In addition, this study underscored that ChatGPT’s susceptibility to variation necessitates periodic revalidation, standardization, and expert oversight. Also, it is concluded that ChatGPT’s role is primarily to complement, rather than supplant, the guideline-based care provided by multidisciplinary teams. Before full clinical deployment can be considered, it is essential to conduct multicenter trials that encompass a more extensive array of items and engage in direct, comparative tracking of the LLM versions.

## Figures and Tables

**Table 1:** Correct/false questions and Likert medians at initial day, 15th day, and 180th day for binary answers.

Group	Category	Initial Day ^(1)^	15th Day ^(2)^	180th Day ^(3)^	*p* ^c^
		*n* (%)	*n* (%)	*n* (%)	
Binary Answers	Correct answers	47 (92.2)	47 (92.2)	50 (98.0)	0.862
	False answers	4 (7.8)	4 (7.8)	1 (2.0)	
	Variable	Median (IQR)	Median (IQR)	Median (IQR)	*p* ^k^/Pairwise Difference
	3-point Likert	2.00 (1.00)	2.00 (1.00)	3.00 (1.00)	0.028 */2 < 3
	6-point Likert	6.00 (1.00)	6.00 (1.00)	6.00 (1.00)	0.496/-

* *p* < 0.05; k = Kruskal–Wallis H-test; c = Chi-square test; ^(1)^ = Day 0, ^(2)^ = Day 15, ^(3)^ = Day 180.

**Table 2:** Variable differences between groups for binary responses.

Likert	Group	Variable	Median	IQR	*p*	Pairwise Difference	Kendall’s W
3-point Likert	Author 1	Initial day ^(1)^	2.00	1.00	0.331	-	0.022
		15th Day ^(2)^	3.00	1.00			
		180th Day ^(3)^	3.00	1.00			
	Author 2	Initial day ^(1)^	2.00	1.00	0.131	-	0.040
		15th Day ^(2)^	2.00	1.00			
		180th Day ^(3)^	3.00	1.00			
	Author 3	Initial day ^(1)^	2.00	1.00	0.711	-	0.007
		15th Day ^(2)^	2.00	1.00			
		180th Day ^(3)^	2.00	1.00			
	Author 4	Initial day ^(1)^	3.00	1.00	0.048 *	1 < 3	0.060
		15th Day ^(2)^	2.00	1.00			
		180th Day ^(3)^	3.00	1.00			
	Author 5	Initial day ^(1)^	3.00	1.00	0.015 *	1 < 3	0.082
		15th Day ^(2)^	2.00	1.00			
		180th Day ^(3)^	3.00	1.00			
6-point Likert	Author 1	Initial day ^(1)^	6.00	1.00	0.307	-	0.023
		15th Day ^(2)^	6.00	1.00			
		180th Day ^(3)^	6.00	1.00			
	Author 2	Initial day ^(1)^	6.00	1.00	0.789	-	0.005
		15th Day ^(2)^	6.00	1.00			
		180th Day ^(3)^	6.00	1.00			
	Author 3	Initial day ^(1)^	6.00	1.00	0.549	-	0.012
		15th Day ^(2)^	5.00	1.00			
		180th Day ^(3)^	5.00	1.00			
	Author 4	Initial day ^(1)^	6.00	1.00	0.789	-	0.005
		15th Day ^(2)^	6.00	1.00			
		180th Day ^(3)^	6.00	1.00			
	Author 5	Initial day ^(1)^	6.00	1.00	0.627	-	0.009
		15th Day ^(2)^	6.00	1.00			
		180th Day ^(3)^	6.00	1.00			

* *p* < 0.05; *p*-values derived from the Friedman test. Pairwise Difference indicates post hoc Wilcoxon signed-rank comparisons (e.g., “1 < 3” = Day 0 vs. Day 180), ^(1)^ = Day 0, ^(2)^ = Day 15, ^(3)^ = Day 180).

**Table 3:** Correct and false questions and Likert medians at initial day, 15th day, and 180th day for multiple-choice answers.

Group	Category	Initial Day ^(1)^	15th Day ^(2)^	180th Day ^(3)^	*p* ^c^
		*n* (%)	*n* (%)	*n* (%)	
Multiple	Correct answers	46 (90.20)	47 (92.20)	43 (84.30)	0.471
	False answers	5 (9.80)	4 (7.80)	8 (15.70)	
	Variable	Median (IQR)	Median (IQR)	Median (IQR)	*p* ^k^/Pairwise Difference
	3-point Likert	2.00 (1.00)	2.00 (1.00)	2.00 (1.00)	0.001 */1 < 2, 3 < 2
	6-point Likert	6.00 (1.00)	6.00 (0.00)	6.00 (1.00)	0.001 */1 < 2, 3 < 2

* *p* < 0.05; k = Kruskal–Wallis H-test, c = Chi-square test, ^(1)^ = Day 0, ^(2)^ = Day 15, ^(3)^ = Day 180.

**Table 4:** Inter-variable differences between groups for multiple-choice answers.

Likert	Group	Variable	Median	IQR	*p*	Pairwise Difference	Kendall’s W
3-point Likert	Author 1	Initial day ^(1)^	2.00	1.00	0.004 *	2 > 3, 2 > 1	0.110
		15th Day ^(2)^	2.00	1.00			
		180th Day ^(3)^	2.00	1.00			
	Author 2	Initial day ^(1)^	2.00	1.00	0.004 *	2 > 3, 2 > 1	0.110
		15th Day ^(2)^	2.00	1.00			
		180th Day ^(3)^	2.00	1.00			
	Author 3	Initial day ^(1)^	2.00	1.00	0.644	-	0.009
		15th Day ^(2)^	2.00	1.00			
		180th Day ^(3)^	2.00	1.00			
	Author 4	Initial day ^(1)^	2.00	1.00	0.005 *	2 > 3, 2 > 1	0.103
		15th Day ^(2)^	2.00	1.00			
		180th Day ^(3)^	2.00	0.00			
	Author 5	Initial day ^(1)^	2.00	1.00	0.137	-	0.039
		15th Day ^(2)^	2.00	1.00			
		180th Day ^(3)^	2.00	1.00			
6-point Likert	Author 1	Initial day ^(1)^	6.00	1.00	0.159	-	0.036
		15th Day ^(2)^	6.00	0.00			
		180th Day ^(3)^	6.00	1.00			
	Author 2	Initial day ^(1)^	6.00	1.00	0.012 *	2 > 1	0.087
		15th Day ^(2)^	6.00	0.00			
		180th Day ^(3)^	6.00	1.00			
	Author 3	Initial day ^(1)^	6.00	1.00	0.043 *	2 > 1	0.062
		15th Day ^(2)^	6.00	1.00			
		180th Day ^(3)^	6.00	1.00			
	Author 4	Initial day ^(1)^	6.00	1.00	0.012 *	2 > 1, 2 > 3	0.087
		15th Day ^(2)^	6.00	0.00			
		180th Day ^(3)^	6.00	1.00			
	Author 5	Initial day ^(1)^	6.00	1.00	0.009 *	2 > 1, 2 > 3	0.092
		15th Day ^(2)^	6.00	0.00			
		180th Day ^(3)^	6.00	1.00			

* *p* < 0.05; *p*-values obtained from the Friedman test. Pairwise Difference lists significant post hoc Wilcoxon signed-rank comparisons between time points ^(1)^ = Day 0, ^(2)^ = Day 15, ^(3)^ = Day 180; the symbols “>” or “<” denote the direction of the median change.

## Data Availability

The dataset is available on request from the authors.

## Abbreviations

IBD	Inflammatory Bowel Disease
UC	Ulcerative Colitis
CD	Crohn’s Disease
AI	Artificial Intelligence
LLM	Large Language Model
ECCO	European Crohn’s and Colitis Organization
ChatGPT	Chat Generative Pretrained Transformer
